# Exploring Product Release from Yeast Cytosine Deaminase with Metadynamics

**DOI:** 10.1021/acs.jpcb.3c07972

**Published:** 2024-03-22

**Authors:** Kayla A. Croney, James McCarty

**Affiliations:** Department of Chemistry, Western Washington University, Bellingham, Washington 98225, United States

## Abstract

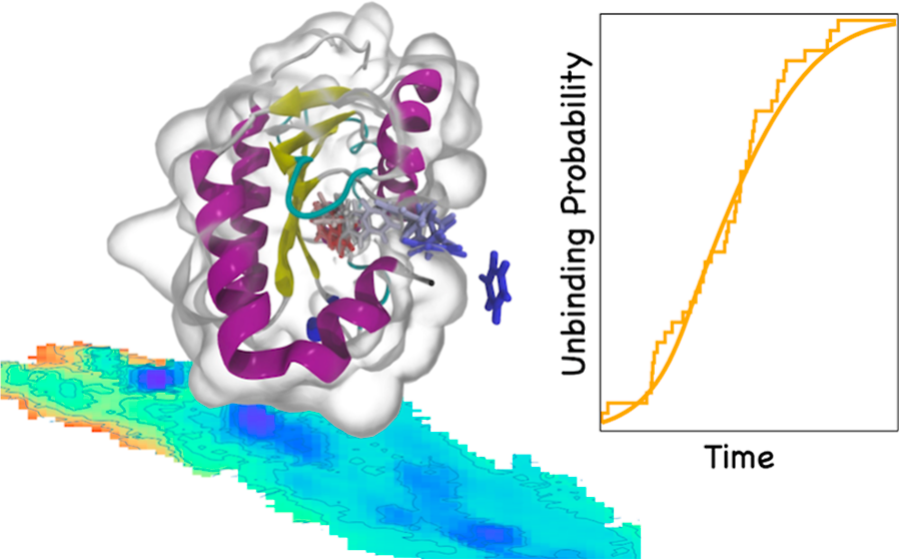

The yeast cytosine deaminase (yCD) enzyme/5-fluorocytosine prodrug system is a promising candidate for targeted chemotherapeutics. After conversion of the prodrug into the toxic chemotherapeutic drug, 5-fluorouracil (5-FU), the slow product release from the enzyme limits the overall catalytic efficiency of the enzyme/prodrug system. Here, we present a computational study of the product release of the anticancer drug, 5-FU, from yCD using metadynamics. We present a comparison of the 5-FU drug to the natural enzyme product, uracil. We use volume-based metadynamics to compute the free energy landscape for product release and show a modest binding affinity for the product to the enzyme, consistent with experiments. Next, we use infrequent metadynamics to estimate the unbiased release rate from Kramers time-dependent rate theory and find a favorable comparison to experiment with a slower rate of product release for the 5-FU system. Our work demonstrates how adaptive sampling methods can be used to study the protein–ligand unbinding process for engineering enzyme/prodrug systems and gives insights into the molecular mechanism of product release for the yCD/5-FU system.

## Introduction

**Figure 1:**
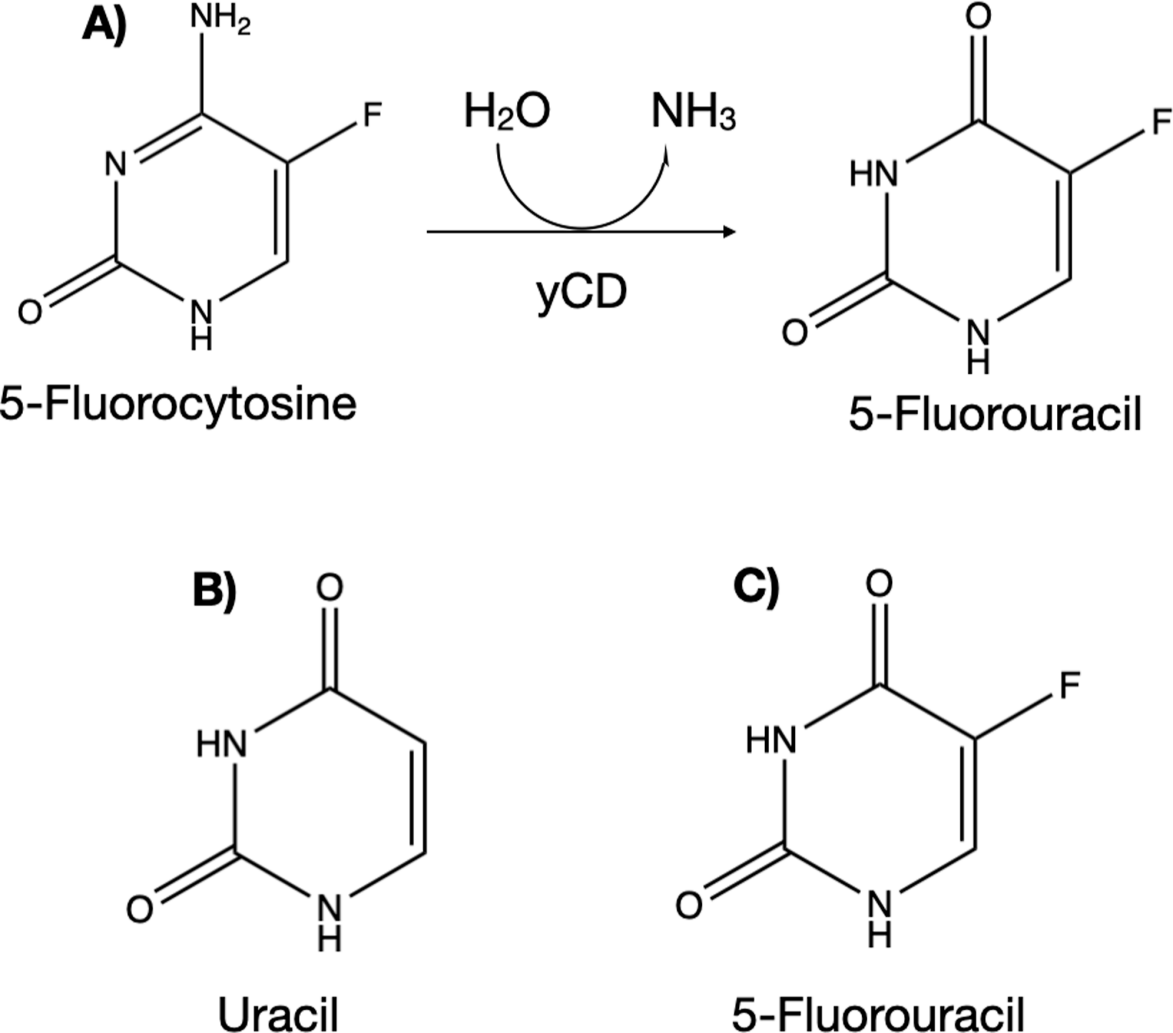
(A) Reaction scheme for the conversion of the prodrug (5-FC) to the anticancer drug 5-FU by the yCD enzyme. (B) 2D structure of the natural enzyme product, uracil, and (C) the anticancer drug, 5-FU.

Prodrugs are chemicals with low inherent toxicity that can be administered and subsequently converted into a toxic chemotherapeutic drug by specific activating enzymes. Genes that encode prodrug-activating enzymes are a promising strategy for tumor therapy. The goal of these therapies is to kill tumor cells by activating a prodrug in the tumor cell, minimizing harm to normal cells that do not express the prodrug-activating enzyme.^1,2^ The yeast cytosine deaminase (yCD) is a zinc metalloenzyme that catalyzes the deamination of cytosine to uracil in the yeast pyrimidine salvage pathway. Cytosine deaminase is of interest in cancer gene therapy because it can convert the prodrug, the antifungal agent 5-fluorocytosine (5-FC) to the anticancer drug 5-fluorouracil (5-FU) (Figure 1).^3−5^ 5-FU is used as a drug to treat colon, pancreatic, and breast cancers, although it also causes gastrointestinal and hematological toxicities.^6,7^ The yCD enzyme is a 35 kDa protein comprising 158 residues and forms a homodimer with each subunit containing a central β-sheet and six α-helices.

By targeting specific mutations, engineered proteins might be designed that improve the overall catalytic turnover of the conversion of 5-FC to 5-FU or that improve the thermal stability of yCD. A potential rate-limiting step is the product release from the active site of yCD, slowing down the overall effectiveness of the drug.^8^ For example, product release and overall catalysis were enhanced by engineering a yCD enzyme with an extended C-terminus having a flexible α-helix to enhance protein conformational dynamics.^9^ A study of the binding/unbinding process of the prodrug/enzyme product, 5-FU, from the yCD enzyme using molecular simulations could help determine key bottlenecks that can be targets for mutations aimed at improving the rate of release of 5-FU out of the active site.

Computational prediction of receptor–ligand binding affinities and binding/unbinding rates is an important step in the design of novel drugs and enzymes.^10−13^ Conformational flexibility is a crucial component in accurately predicting thermodynamic and kinetic properties, and atomistic molecular dynamics (MD) simulations can play a significant role in revealing how protein dynamics modulates receptor–ligand interactions.^14^ However, atomistic MD simulations in an explicit solvent are limited by the statistical sampling that can be obtained at a reasonable computational cost. The residence time for a drug buried in the active site can be orders of magnitude longer than the simulation time. For this reason, ligand (un)binding can be seen as a rare event, and a number of different enhanced sampling methods have been developed to provide the free energy profile or association/dissociation rates.^15−19^

Several previous computational studies have investigated product release from yCD. Steered MD (SMD) was used to explore ligand release from yCD, suggesting multiple unbinding paths and that the C-terminal helix is critical for ligand release.^20^ More recently, random acceleration molecular dynamics was used to sample distinct unbinding pathways for the product release of 5-FU from yCD.^21^ Together with recent QM/MM simulations,^22^ this prior work provides mechanistic insights into the catalytic cycle of the yCD system. The suggestion that product release is a rate-determining step implies that there is some affinity between the drug 5-FU and the yCD protein, and its release from the active site requires overcoming a kinetic barrier indicative of a rare event.

In the present work, we use metadynamics^23^ in combination with atomistic MD simulations to compare the escape of both uracil and the chemotherapeutic drug, 5-FU, from the yCD dimer active site. In metadynamics, a history-dependent bias is added to relevant collective variables (CVs) in order to enhance fluctuations along slow degrees of freedom.^24^ Recently, a volume-based metadynamics approach was introduced as an efficient way to completely sample ligand unbinding events, enabling the characterization of unbinding paths and the computation of the free energy surface (FES) along low-dimensional reaction coordinates.^25^ The method has been applied in a detailed study of mono-ADP-ribose (ADPr) in complex with mono-ADP-ribosylation hydrolases, for which conformational dynamics and induced-fit effects are significant.^26^ Here, we perform volume-based metadynamics simulations to comprehensively sample the product release from the yCD dimer active site and compute a FES for both the uracil/yCD and 5-FU/yCD systems. This allows us to compare the thermodynamics of product release for the natural product (uracil) and drug (5-FU) and identify differences in the FES. We analyze the different unbinding paths for interactions between ligand and protein that are important for product release. Next, we turn to an analysis of the kinetics of unbinding. Unbiased estimates of dissociation rate constants, *k*_off_, can be estimated by performing metadynamics with a slow deposition stride, so-called infrequent metadynamics.^27^ The idea is that the time-dependent bias potential accumulates slow enough to push the drug out of the active site without perturbing the transition state ensemble. The reliability of the times predicted from metadynamics can be assessed by considering the statistics of a Poisson process.^28^ We make use of a recent method based on Kramers’ rate theory for calculating the barrier crossing rate from time-dependent biased simulations.^29^ We present a comparison of the *k*_off_ rates for uracil and 5-FU and find that product release of uracil is an order of magnitude faster than the 5-FU drug. Our computational work corroborates experimental evidence of weak binding affinity for the product with yCD and product release as the rate-limiting step.

## Materials and Methods

### System Preparation and Molecular Dynamics Simulation

**Figure 2:**
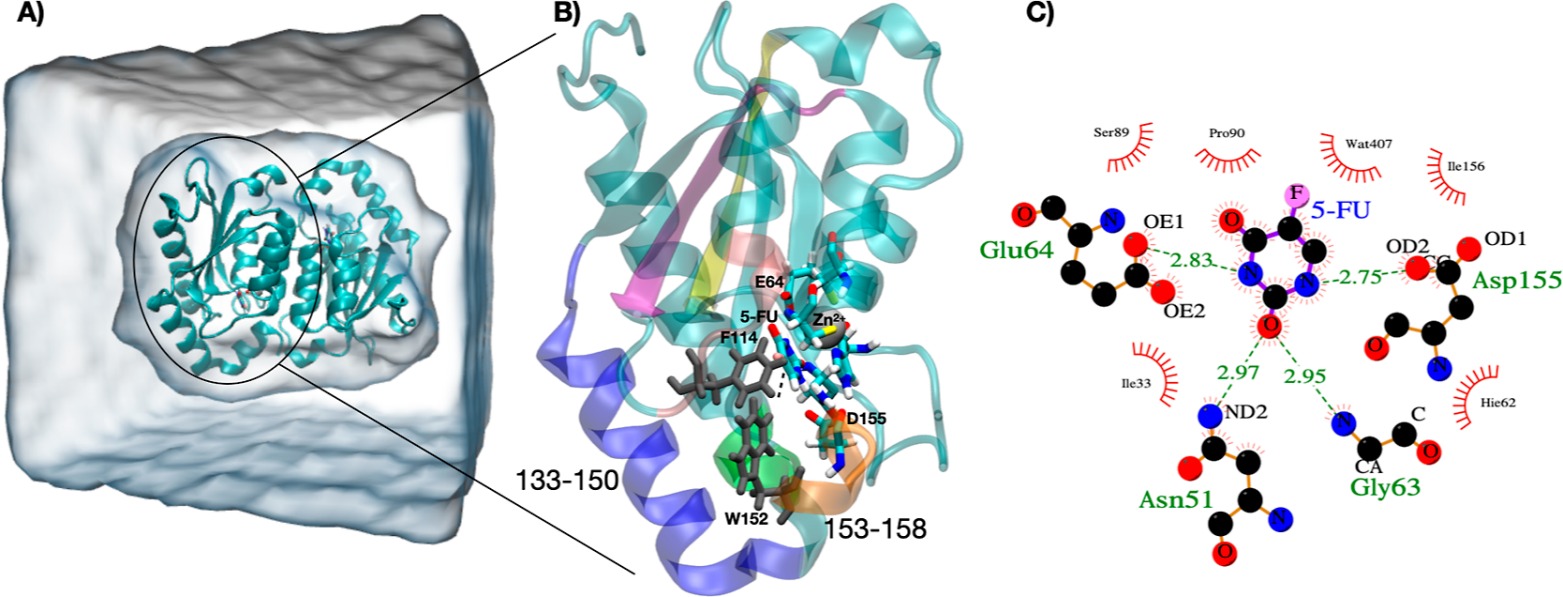
(A) Homodimer system complexed with 5-FU solvated in a cubic box. The active site contains a Zn^2+^ ion coordinated with His62, Cys91, Cys94, and a water molecule. B) Cartoon representation of the yCD monomer with residues 133–150 colored in blue and residues 153–158 colored in orange representing the C-terminus. Additionally, residues 26–31 are colored in green, residues 103–110 in purple, residues 82–87 in yellow, and 114–119 in pink. Two side chains important for product release are Trp152 and Phe114, which are shown in gray. Side chains Glu64 and Asp155, which form hydrogen bonds with the ligand, are also labeled. C) 2D plot of the molecular interactions between yCD and the 5-FU drug in the active site.

The starting structure was prepared from the crystal structure of yCD complexed with a transition state analogue 4(R)-hydroxyl-3,4-dihydropyrimidine (PDB code: 1P6O).^30^ The inhibitor in the active site was replaced with either uracil or 5-FU, and a zinc-coordinated water molecule was added to the active site.^31^ Simulations were performed on the homodimer system in a box with periodic boundary conditions solvated with 10,552 TIP3P water molecules with a minimal distance of 14 Å between the solute and the edge of the box. The final system contained 36,612 atoms in a 7.91 nm × 7.79 nm × 7.36 nm box (see Figure 2A). Figure 2B shows one monomer highlighting the active site. All protein atoms were described by the Amber14SB force fields.^32^ The uracil and 5-FU molecules were described by the general AMBER force fields^33^ with partial atomic charges determined using the restrained electrostatic potential (RESP) model^34^ at the B3LYP/6-31G* level of theory. Quantum chemistry calculations were performed using GAMESS^35^ with RESP fitting performed using Multiwfn.^36^ The ligand was built and parametrized using antechamber.^37^ For the zinc ion in the active site, we used the Lennard-Jones 6–12 nonbonded parameter set for Zn^2+^ in TIP3P water from Li et al.^38^ Additional sodium and chloride counterions were added to neutralize the charge of the whole system and to achieve a physiological salt concentration of 150 mM. The system was prepared using the AmberTools21 software^39^ and converted to GROMACS topology and coordinates file format using ACPYPE.^40^

All simulations were performed using GROMACS 2019.4^41^ patched with PLUMED 2.4.^42,43^ A steepest descent energy minimization was performed until the maximum force was ≤1000 kJ/mol/nm. After energy minimization, the system was first equilibrated for 2 ns with position restraints on all protein heavy atoms in the *NVT* ensemble at 300 K using the stochastic velocity rescaling thermostat.^44^ The solvent and the solute were coupled to separate heat baths. This was followed by a 15 ns equilibration in the *NPT* ensemble at 1 bar without position restraints using a Parrinello–Rahman barostat.^45^ Metadynamics simulations were run for 500 ns in the *NPT* ensemble. We used an integration time step of 2 fs. All bonds to hydrogen atoms were constrained using the LINCS algorithm.^46^ Long-range electrostatic interactions were calculated with the particle mesh Ewald algorithm^47^ using a grid spacing of 0.16 nm and a cutoff of 1.0 nm for both the real-space Coulombic and Lennard-Jones interactions.

### Volume-Based Metadynamics

Well-tempered metadynamics^48^ was performed using a volume-based approach that uses the spherical coordinate system as CVs as described recently.^25^ One CV is the radial distance, ρ, between the center of mass of the ligand and protein. The other two CVs are its polar angle, ϕ, measured from the *z*-axis, and the azimuthal angle, θ, from its projection on the *x*–*y* plane (see Figure S1 for a depiction of the volume-based CVs). In volume-based metadynamics, the ligand is confined to a spherical region with radius ρ_s_, larger than the radius of gyration of the protein, by a restraining potential
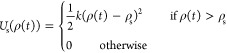
1with *k* = 200 kJ/mol/Å^2^ and ρ_s_ = 28 Å is the radius of the spherical restraint. ρ(*t*) is the radial distance of the ligand from the center of mass of the target protein at time *t*. This approach is an extension of funnel metadynamics,^13^ where a funnel-shaped potential limits the volume explored by the unbound ligand in solution. Well-tempered metadynamics was performed with an initial hill height of 1.2 kJ/mol and hills deposited every 1 ps with a bias factor set to γ = 20. The Gaussian widths, σ, was set to 1 Å, π/16 rad, and π/8 rad, for ρ, θ, and ϕ, respectively. The coordinates were aligned to the heavy atoms of a reference frame, wrapping the ligand around the protein so they remain in the same periodic image. Because the system is a homodimer, a harmonic constraint is added to the RMSD of the second protein chain with bound ligand in the active site to allow us to study product release from only one of the dimer subunits.

Reweighting is performed using the time-independent free energy estimator of Tiwary and Parrinello.^49^ The free energy surface is projected along two dimensions: the radial distance between the center of mass of the ligand and protein, ρ, and the coordination number, *C*_N_, between the ligand and host protein
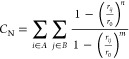
2where *r*_*ij*_ is the distance between atom *i* and *j*; group A is the set of all non-hydrogen atoms in the ligand, and group B is the set of all non-hydrogen atoms in the protein plus the zinc ion in the active site. We set the cutoff to define a native contact as *r*_0_ = 4.5 Å with *m* = 6 and *n* = 12 as the exponents in the switching function.

### Infrequent Metadynamics and Path Collective Variables

For computing kinetic rate constants, we performed a separate set of simulations using infrequent metadynamics^27^ (iMetaD). We used two CVs for these iMetaD simulations. The first CV was a path CV in contact map space, defined as a distance, *s*, along a reference path from the bound state to the fully solvated unbound state.^50^ The path CV formalism is useful for studying the ligand unbinding process, and it has been used to compute free energy surfaces and kinetic behavior.^51−53^ The distance along the unbinding path was defined in a contact map space. A contact map was first constructed from identifying relevant interactions along the unbinding trajectory from our previous volume-based metadynamics simulations. To construct a path CV in contact map space, we used 8 frames starting from the bound state and ending in the unbound state, generated from the volume based metadynamics trajectory. The initially selected frames were adjusted in order to make them equally spaced using the *pathtools* command line tool in PLUMED2. The progress along the path, *s*, and the distance from the path, *z*, are calculated using the geometric formulation of Díaz Leines and Ensing.^54^ In addition to the path CV, we also biased along the distance between the center of mass of the ligand and the center of mass of the active site. We performed well-tempered metadynamics on the contact map path, *s*, and the distance CV with Gaussian widths of 0.13 and 0.02 nm, respectively. Gaussians were deposited every 100 ps, with a starting height of 1.5 kJ/mol and a bias factor of 15. Table S1 provides a list of the atom pairs that form the contacts used in the contact map path CV and the parameters used for the switching function.

To verify the transition from the bound state to the unbound state, we also monitored (without biasing it) the time evolution of important contacts between the ligand and side chains in the binding pocket. This CV is defined as the coordination number analogous to eq 2, with group A being the nitrogen and oxygen atoms on the ligand and group B being the OE1, OE2 atoms on Glu64, the ND2 atom on Asn51, the OD1, OD2 atoms on Asp155, and the Zn^2+^ ion in the active site. These interactions are shown in the 2D plot in Figure 2C. We define a crossing event from the fully bound state to the fully unbound state when this coordination CV reaches a value of less than 0.01.

### Kramers’ Time-Dependent Rate Theory

To obtain transition rates from iMetaD simulations, we use the recent method based on Kramers’ time-dependent rate (KTR) theory.^29^ This procedure has been shown to yield better rates for suboptimal CVs since the quality of the bias can be extracted from the fitting procedure. Briefly, the survival probability, *S*(*t*), after some time *t* is adapted from the methods used to analyze single-molecule pulling experiments^55^
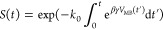
3where β = 1/*k*_B_*T*. *V*_MB_ is the time-dependent maximum bias (MB) averaged over multiple unbinding trajectories^29^
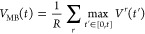
4where *V*^*r*^(*t*) is the instantaneous bias for simulation *r* at time *t* and *R* is the total number of runs. The two remaining unknowns in eq 3 are the unbiased transition rate *k*_0_ and γ, a parameter that is a measure of the quality of the bias. The parameters *k*_0_ and γ are determined by maximizing the likelihood function^29^
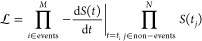
5where *M* is the number of crossing events observed and *N* is the number of nonevents observed up to time *t*_*j*_. In the procedure of Palacio-Rodriguez et al.,^29^ the numerical *V*_MB_(*t*) can either be fit to a spline or modeled with an analytical expression, assuming that the metadynamics bias grows with time in a well-defined manner. In calculating the numerical *V*_MB_ from eq 4, only trajectories that are still in the initial basin at time *t* and thus have not crossed the barrier are included in the sum. This is consistent with the assumption of a continually growing bias analogous to a pulling experiment and means that the metadynamics bias is steadily increasing. Trajectories that have already crossed out of the initial basin at time *t* and hence no longer have an increasing *V*^*r*^(*t*′) are not included in the sum. We fit the numerical value of *V*_MB_ to the form *V*_MB_ = *a* log (1 + *bt*) up until a cutoff time after which time, *V*_MB_ is considered constant at approximately the barrier height. Once *V*_MB_ is modeled, the unknown quantities *k*_0_ and γ in eq 3 are found from numerical maximization of  using the script provided in ref (29).

The quality of the fit can be assessed with the Kolmogorov–Smirnov test (KS-test).^28^ This test compares the empirical cumulative distribution function (ECDF), computed as a step function that counts discrete jump events, to the theoretical cumulative distribution function (TCDF). The TCDF is calculated as 1 – *S*(*t*), with the survival function, *S*(*t*), given by eq 3, using the model for *V*_MB_(*t*) and the fitted parameters *k*_0_ and γ. We compare the ECDF obtained from the accumulated crossing times from iMetaD simulations with the TCDF, extracting a *p*-value to assess if the KS-test passed or not. Uncertainties are estimated from 50 bootstrap samples that pass the KS-test. The simulated crossing times were resampled with replacement, and the values of the 30th and 70th percentiles of the bootstrap samples were used to assess the variance in the estimated *k*_off_ values.

## Results and Discussion

### Thermodynamics of Product Release from yCD

**Figure 3:**
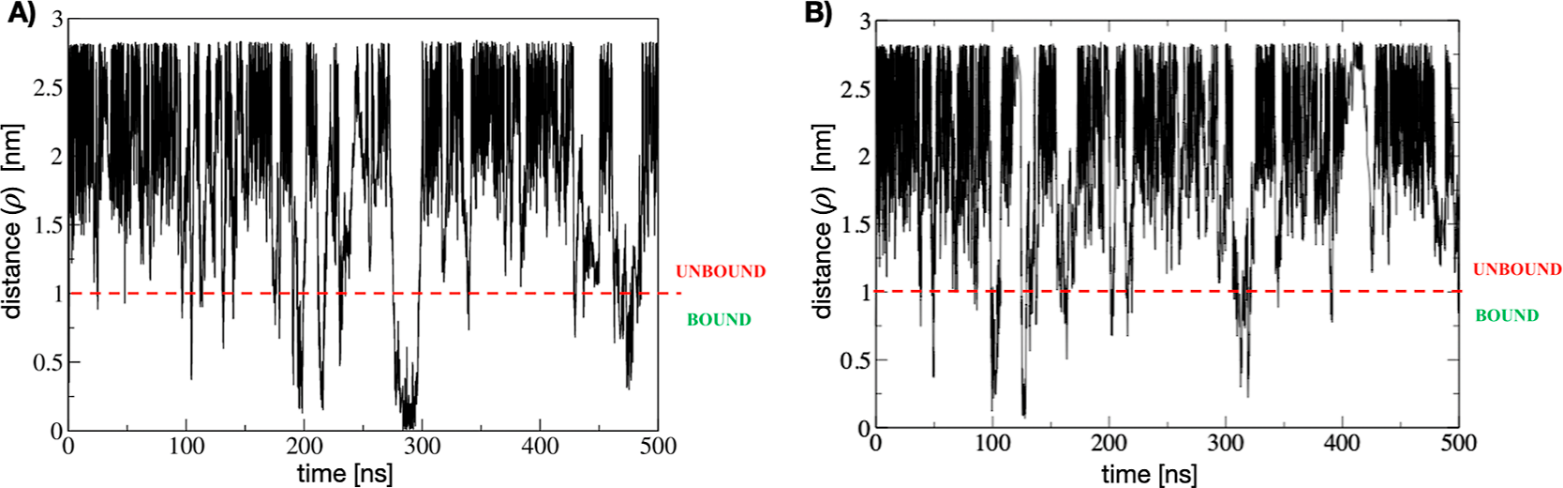
Time series of the distance between centers of mass of (A) 5-FU and (B) uracil from the metadynamics trajectory. Simulations were started in the bound state. Several recrossing events between bound and unbound states are observed.

**Figure 4:**
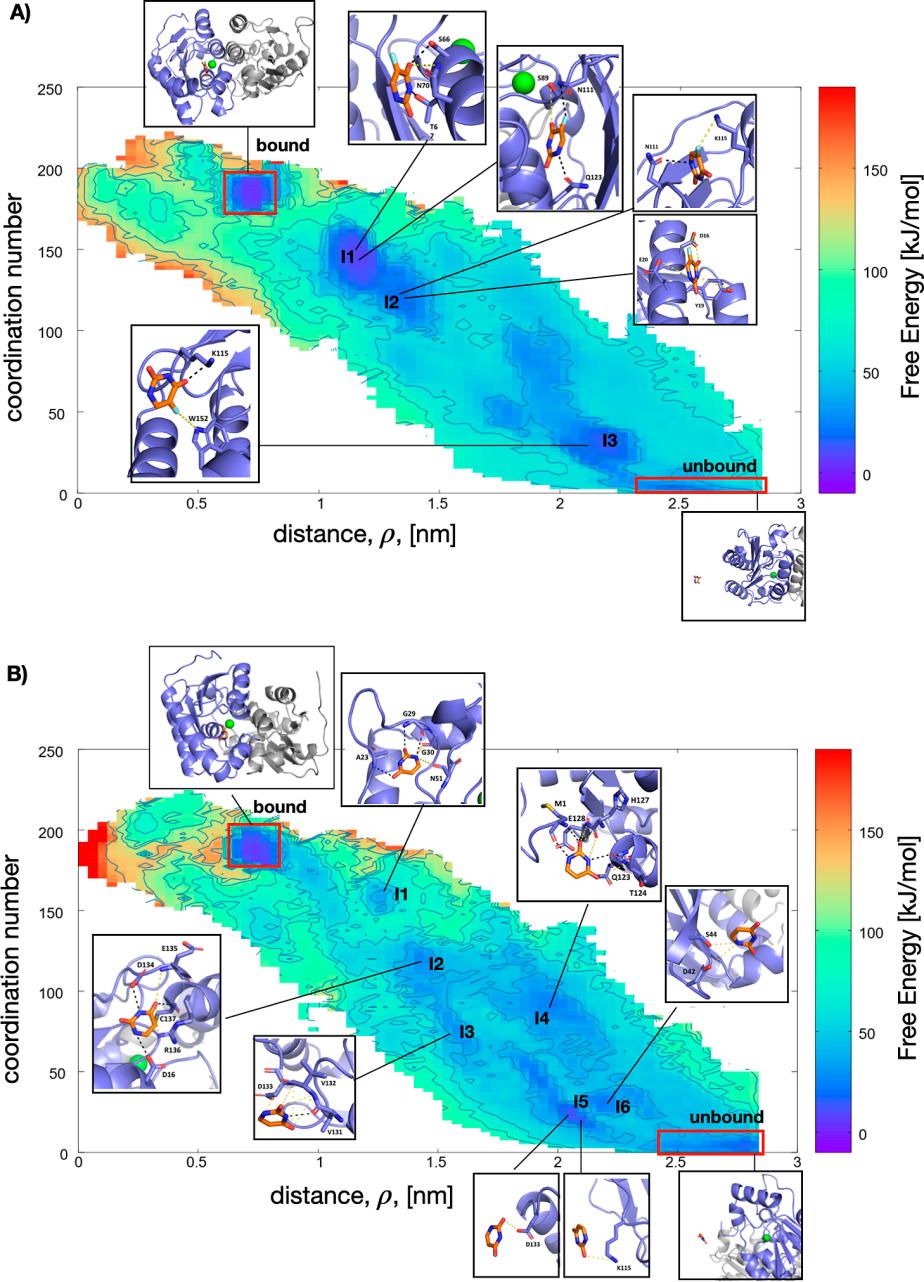
(A) Free energy surface for the product release of the 5-FU drug from the yCD enzyme. The surface is obtained by reweighting the bias along the coordination number CV and distance between center of masses of the ligand and target, ρ. The red boxes highlight the fully bound position and the fully unbound position. Intermediate states along the unbinding path are indicated with I1–I3 along with representative 3D conformations. (B) Free energy surface for the yCD/uracil system with intermediates labeled I1–I6 shown with representative conformations.

We performed production runs of 500 ns MD simulations using volume-based metadynamics for both the 5-FU prodrug/enzyme product and uracil (natural product) complexed with yCD. Figure 3A,B presents the time-series of the center of mass distance between ligand and protein, showing recrossing events between bound and unbound states for 5-FU and uracil, respectively. All volume-based MetaD simulations were started with the ligand in the bound state. Another set of independent simulations was performed to assess convergence and reliability of the calculated free energies. The time-series of these repeated trajectories is presented in Figure S2. The reweighted FES is shown in Figure 4A for the 5-FU system and in Figure 4B for the uracil system. Both free energy surfaces are qualitatively similar, exhibiting a fully bound state and fully unbound state (see red box in Figure 4), along with several intermediate, partially bound states. These intermediate states along the unbinding path are labeled (I1, I2, etc.) with representative structures. The intermediate states for the 5-FU ligand are more pronounced than for the uracil, suggesting the simulation spends more time in these partially bound states for the 5-FU system.

From the reweighted FES, the difference in free energy between the bound and unbound states, obtained by metadynamics, Δ*G*_MetaD_ is
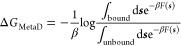
6where β = 1/*k*_B_*T*, **s** are the CVs (ρ and *C*_N_), and *F*(**s**) is the free energy surface. The integrations are performed over two regions in CV space that correspond to the bound state and the unbound state. The integrations are performed numerically on a grid. For uracil, the bound state was defined in the area of the FES in the interval ρ ∈ [0.64 nm, 0.84 nm] and *C*_N_ ∈ [175, 197]. The unbound state is defined as the area of the FES in the interval ρ ∈ [2.4 nm, 2.88 nm] and *C*_N_ ∈ [0, 9.4]. For 5-FU, the bound state was defined in the interval ρ ∈ [0.64 nm, 0.76 nm] and *C*_N_ ∈ [175, 194.9], and the unbound state is defined in the interval ρ ∈ [2.36 nm, 2.68 nm] and *C*_N_ ∈ [0, 5.94]. The convergence of Δ*G*_MetaD_ calculated from eq 6 as a function of the simulation time is presented in Figure S3. Error bars are obtained by the usual block analysis technique. To assess the robustness of these results, Δ*G*_MetaD_ was compared from two independent 500 ns trajectories.

The restraining potential that limits the volume explored by the unbound ligand affects the translational entropy of the unbound state. As described in previous work,^16,25,26^ the correction to the binding free energy is given by
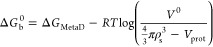
7where *R* is the gas constant, *T* is the temperature, *V*^0^ = 1660 Å^3^ is the inverse of the standard concentration (*C*^0^), and  is the volume of the sphere defined by the restraining potential. *V*_prot_ is the volume of the protein inside the sphere and is estimated using the Mol_Volume code from VMD^56^ with a probe radius of 0.5 Å, a grid step of 0.5 Å, and all van der Waals radii set to 1.7 Å. Table 1 presents a comparison of the thermodynamics of binding uracil or 5-FU with yCD. Comparing the absolute binding affinities, Δ*G*_b_^0^, of uracil and 5-FU, we observe that both ligands have comparable binding affinities, which is not surprising given the similarities of the ligand. The slightly greater affinity calculated for the 5-FU drug with the enzyme may be partially responsible for the lower catalytic efficiency observed in experiments.^8^ The predicted binding free energies correspond to *K*_d_ values in the range of 0.2–0.4 mM, indicating a modest to weak binding affinity (negative Δ*G*_b_°) and that the product release is endergonic, in agreement with experiment.^8^ Independent simulations give Δ*G*_b_° values within 1 kcal/mol.

**Table 1:** Predicted Thermodynamics of Binding for the 5-FU/yCD and Uracil/yCD System

simulation (500 ns)	ligand	Δ*G*_MetaD_ (kcal/mol)	*V*_prot_ (Å^3^)	*T*Δ*S* (kcal/mol)	Δ*G*_b_° (kcal/mol)
1	5-FU	–3.3 ± 0.2	48300	1.9	–5.2 ± 0.2
2	uracil	–2.8 ± 0.2	48300	1.9	–4.7 ± 0.2
3	5-FU	–3.1 ± 0.2	48300	1.9	–5.1 ± 0.2
4	uracil	–2.3 ± 0.3	48300	1.9	–4.2 ± 0.3

### Unbinding Pathways

**Figure 5:**
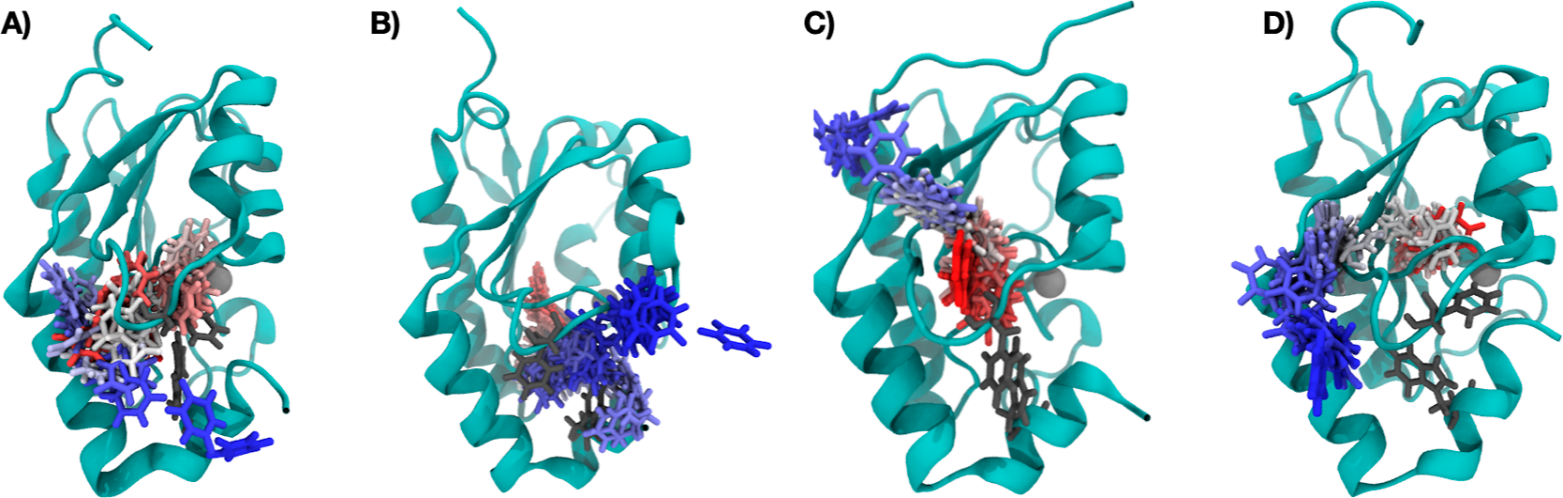
Observed product release pathways (A–D) of 5-FU from yCD sampled during the volume-based MetaD trajectory. The 5-FU drug is colored from red to blue on passing from the bound to the unbound state. The aromatic side chains Phe114 and Trp152 are colored in gray.

We analyze the trajectory by visual inspection to understand the observed product release pathways. These pathways are not weighted by probability, but they are simply representative of the sampled pathways during the metadynamics simulation. Four product release paths (labeled A–D) are shown in Figure 5 for the 5-FU ligand. Most of the unbinding paths involve the C-terminus helix and the loop that contains Phe114, consistent with prior MD simulations.^20^ These observed unbinding pathways are in general agreement with the most frequent pathways observed in prior random acceleration MD simulations (RAMD) of uracil.^21^ In pathway A, the ligand interacts with the α-helix resides 133–150 (shown in blue in Figure 2B). The ligand also interacts with the aromatic Phe114 and Trp152 residues (shown in gray), possibly forming π–π interactions before escape. In pathway B, the ligand exits between the 111–117 loop and the C-terminus residues 153–158 (shown in orange in Figure 2B). During product release, there is rotation of the Phe114 and Trp152, and the ligand still interacts with these aromatic residues while unbinding but does not interact with α-helix resides 133–150. In a third pathway C, the ligand escapes between the residues that form the 111–117 loop. Finally, in pathway D, the product is released between the parallel β sheet formed by residues 103–109 and residues 82–87.

**Figure 6:**
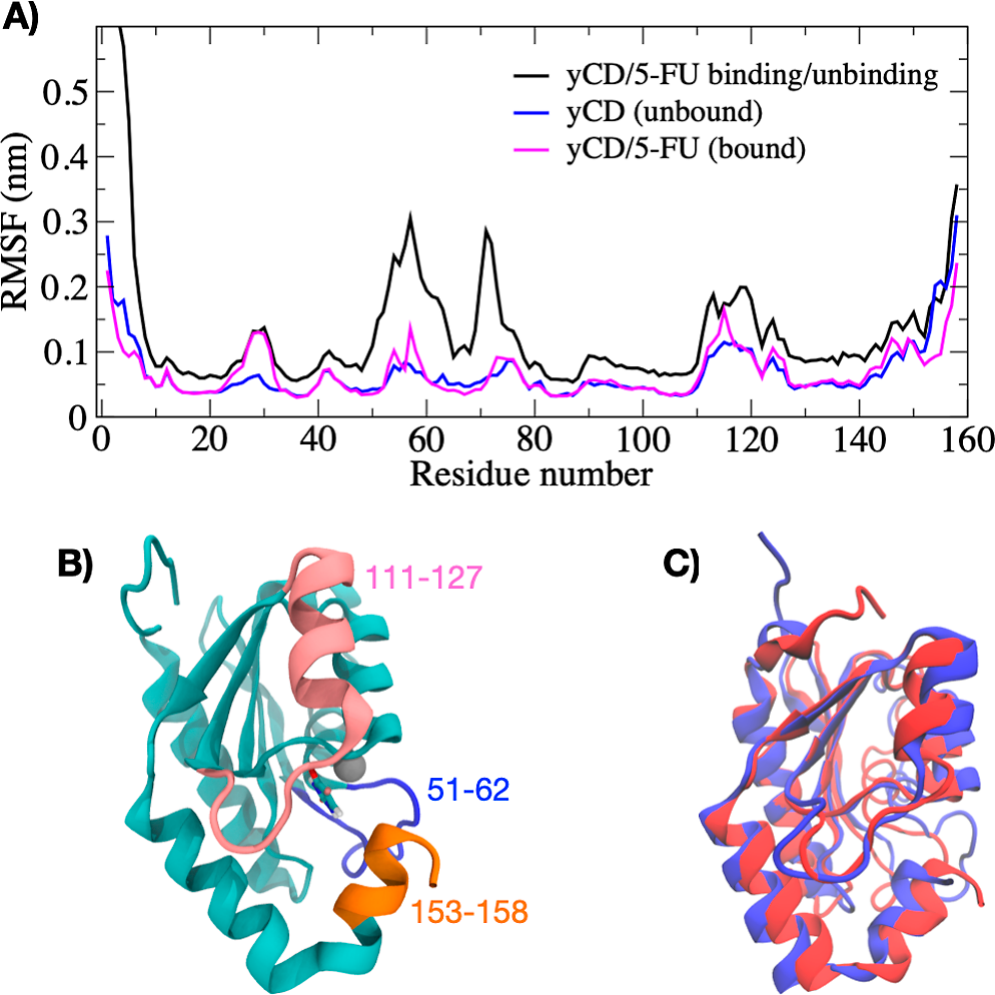
((A) RMSFs of residues during the volume-based metadynamics simulation for the yCD/5-FU (black), yCD in the unbound state (blue), and yCD/5-FU in the bound state (pink) from unbiased simulation. During binding/unbinding, the 5-FU interacts with the 51−61 loop, the 114−119 loop, and the C-terminus. (B) Representative structure of yCD showing regions of enhanced fluctuations. Residues 51–61 are colored in blue, residues 111–127 are colored in pink, and residues 153–158 are colored in orange. (C) Overlay of representative structures of the bound (red) and unbound (blue) conformation showing the motion of the C-terminus and loop 111–117 regions.

Figure 6A shows the root-mean-square fluctuations (RMSF) of the protein over the 500 ns metadynamics 5-FU binding/unbinding trajectory as compared to the RMSF of the protein in the bound state and unbound state, computed from separate unbiased 500 ns MD trajectories. The protein exhibits increased fluctuations during the binding/unbinding simulations in the 51–61 loop region and residues 111–127, which include the 114–119 loop. During the volume-based MetaD simulation, the 5-FU drug interacts with these regions during binding and unbinding, leading to increased fluctuations. All three trajectories exhibit modest flexibility at the C-terminus (residues 153–158) and the 114–119 loop that involves product release. Figure 6B shows a representative structure with the flexible 51–61 loop colored in blue, the flexible residues 111–127 colored in pink, and the C-terminus residues 153–158 colored in orange. Figure 6C compares representative structures from the protein in the bound and unbound states taken from the volume-based MetaD trajectory. We do not observe a large structural difference between the bound and unbound states as shown in Figure 6C, and large scale motion of the C-terminus lid is not necessary for product release in our simulations.

### Kinetics of Product Release from yCD

**Figure 7:**
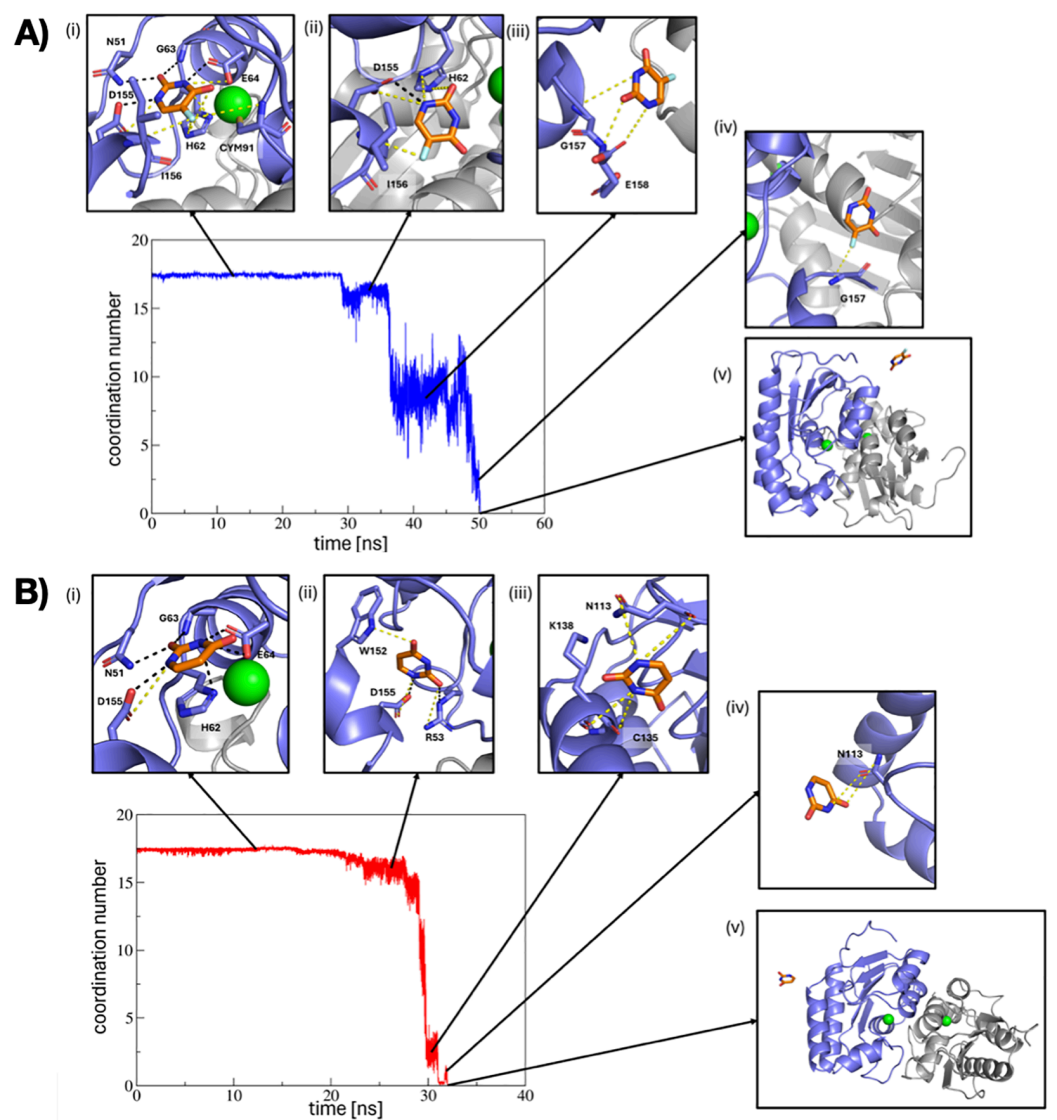
(A) Representative product release iMetaD trajectory for 5-FU showing initial breaking of bound contacts, followed by several long-lived intermediate states before full product release. (B) Representative iMetaD trajectory of product release for uracil. After initial breaking of bound contacts, the uracil fully unbinds without spending much time in intermediate states.

To compare the product release rates of the natural product, uracil, with the drug, 5-FU, we performed iMetaD simulations with a bias deposition stride of 100 ps. We performed 41 independent simulations for each system starting from the bound state and measured the simulation time of the final dissociation event. Starting structures were obtained by selecting frames every 200 ps from the final 10 ns of an unbiased simulation. Each structure was energy minimized and equilibrated for 5 ns in the *NVT* ensemble with random initial velocities drawn from a Maxwell–Boltzmann distribution. An additional 15 ns equilibration in the *NPT* ensemble was performed before initiating metadynamics. During the iMetaD simulation, the coordination CV was used to monitor the unbinding progress. The final unbound state was determined when the coordination CV reached a value of less than 0.01. However, as shown in Figure 7, product release involves several intermediate states before the final unbound state is reached. Figure 7A shows the long-lived intermediate, partially unbound states for the 5-FU unbinding. The first step is the breaking of the stable contacts between the drug and Gly63, Asp155, Glu64, and Asn51. However, after these contacts break, the drug spends time in intermediate states, indicating that the 5-FU interacts with side chains while being released. As a comparison, Figure 7B shows a representative unbinding trajectory for the uracil molecule. Similarly, the initial release involves the breaking of contacts in the bound state; however, once these contacts break, the molecule leaves the binding site with relative ease.

**Figure 8:**
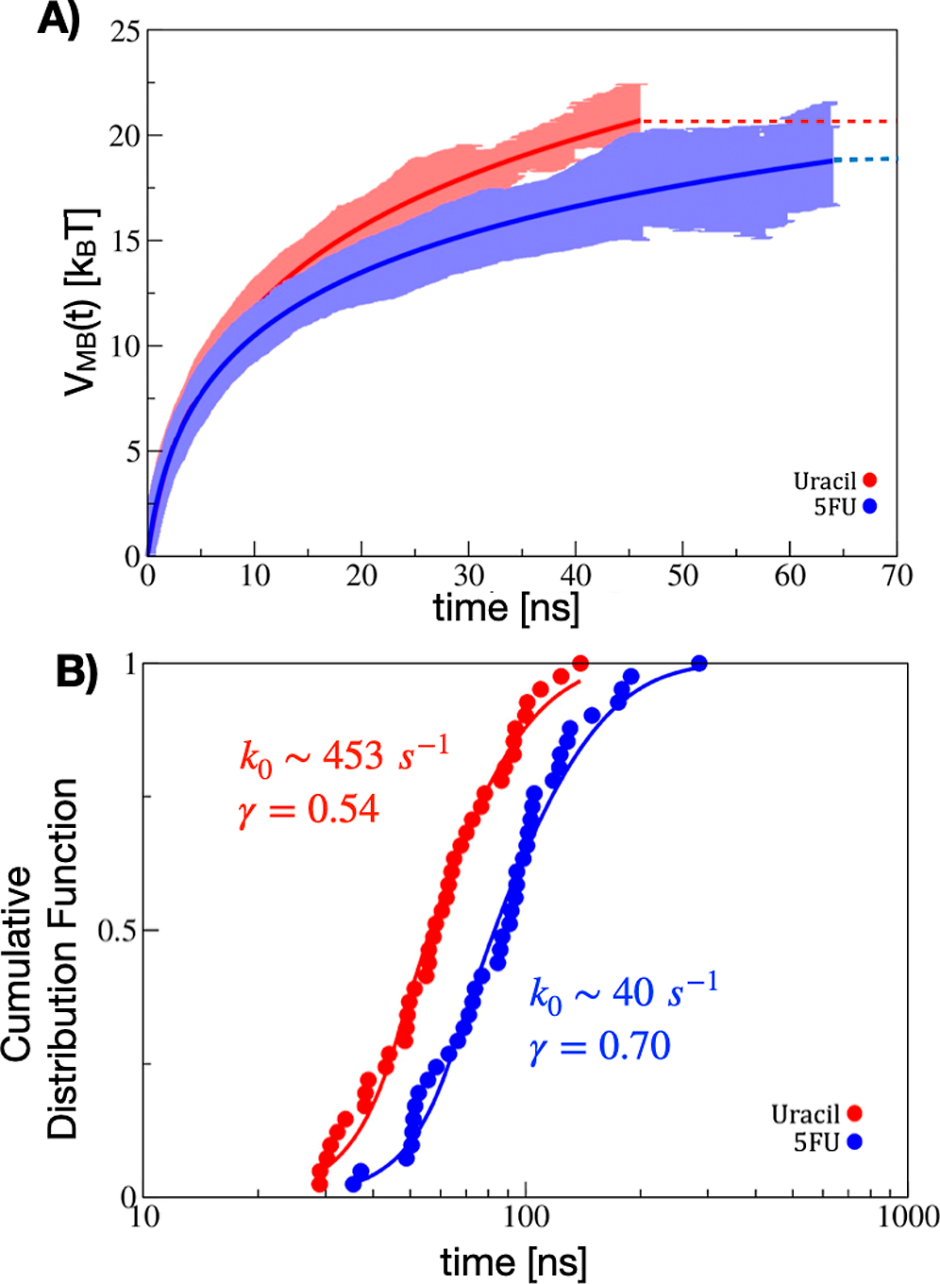
(A) Average max metadynamics bias (*V*_MB_) as a function of time for 5-FU (blue) and for uracil (red). The line is fit to a logarithmic function. The shaded region corresponds to the standard deviation over the trajectories. The dashed line indicates the extrapolation of *V*_MB_ to a fixed upper limit. (B) ECDF of unbinding times (points) from 41 independent biased simulations for both 5-FU (blue) and uracil (red). The time in the *x*-axis is the biased simulation time. The solid line is the theoretical cumulative distribution that is fit to the data using Kramers’ rate theory with fit parameters *k*_0_ and γ.

We performed 41 independent iMetaD simulations for both the yCD/5-FU and yCD/uracil system to construct the cumulative distribution of escape times. Figure 8A shows the accumulated average max bias, *V*_MB_, as a function of the simulation time with the shaded region representing the standard deviation. The average of the max bias is computed only for trajectories that remain in the bound state so that the average max bias is steadily increasing. The numerical *V*_MB_(*t*) is fit to a logarithmic function up until a cutoff point (shown by the dashed lines in Figure 8A). The value of *V*_MB_ approaches a stationary level that should in principle correspond to the true free energy barrier in the case of an ideal CV. This cutoff time after which the model for *V*_MB_ is considered to have reached a stationary value is chosen to maximize the overall quality of the fit (*p*-value) and minimize the KS test statistic (*D*) of the fit to the empirical *S*(*t*). This procedure provides the best possible fit of the model for *V*_MB_ to the empirical ECDF from the simulation data.

Figure 8B shows the ECDF of unbinding times from the 41 iMetaD trajectories and the corresponding best fit to the TCDF using eq 3, found by maximizing the likelihood function. The ECDF is computed directly from the accumulated biased simulations without rescaling the time. The unbiased rate is estimated by fitting the ECDF directly using the KTR theory. The *p*-values for the best fit of the model to the ECDF data are 0.86 for the yCD/5-FU system and 0.97 for the yCD/uracil system. The fit yields an estimate of the unbiased rate, *k*_0_, and the value of γ, which assess the quality of the CVs used. These values are presented in Table 2 for both the 5-FU and uracil system. Consistent with the overall slightly more favorable binding affinity for 5-FU, the calculated unbinding rate is *k*_off_ ∼ 40 s^–1^ for 5-FU and *k*_off_ ∼ 453 s^–1^ for uracil. The relative rate of unbinding is ∼10 times greater for uracil. The fit to the ECDF data is extremely sensitive to the estimate of *V*_MB_, and we expect large uncertainties due to the relatively small number of samples. To give an idea of the variance in the estimated product release rates, we performed a bootstrap analysis, resampling the escape times with replacement. Table 2 reports the values of the 30th and 70th percentiles of the bootstrap samples that passed the KS-test. While there is a significant variance in the estimated rates, the reported best-fit estimate for *k*_off_ is within this range.

**Table 2:** Product Release Rates of 5-FU and Uracil from Infrequent Metadynamics

ligand	γ	koff (s^–1^)	lower 30th percentile (s^–1^)	upper 70th percentile (s^–1^)	*p*-value
5-FU	0.70	40	3.3	226	0.86
Uracil	0.54	453	186	992	0.97

The hyperdynamics approach of rescaling the biased simulation time is typically used to obtain kinetics from infrequent metadynamics.^27^ Even with a Gaussian deposition stride of 100 ps, a fit of the rescaled cumulative distribution function fails the KS-test, indicating that the CVs that we employed were not optimal. The fit to the ECDF using the hyperdynamics approach is shown in Figure S4.

## Conclusions

We have used metadynamics simulations to study the product release of both 5-FU and uracil from the yCD enzyme. This computational study corroborates experimental and computational work suggesting that product release is the rate-limiting step. We performed volume-based metadynamics to sample the ligand/enzyme conformational space and found that the product release is not spontaneous with a change in free energy of ∼5 kcal/mol, corresponding to a *K*_d_ value in the 0.2 mM range. This would indicate modest association of the 5-FU with the yCD enzyme and slow exchange of product. This is in agreement with prior umbrella sampling simulations along one of the reaction coordinates that estimated a free energy cost of product release of ∼6.8–7.0 kcal/mol.^21^ Experimental NMR studies estimated a *K*_d_ value for the binary complex of ∼20 mM, which is higher than our estimate but in qualitative agreement.^8^

The MetaD simulations suggest two possible origins for the slow product release. First is the interaction of the aromatic uracil or 5-FU with the Zn^2+^ ion in the bound state. This interaction, along with interactions between the ligand and the side chains Glu64, Asp155, Gly63, and Asn51 in the active site, must be broken before product release. The observation of long-lived, partially bound metastable intermediate states for the 5-FU system suggests an origin for the slower dissociation rate of 5-FU as compared with uracil. We observe several unbinding events among residues 153–158 (C-terminus) and among the aromatic side chains Trp152 and Phe114 in the 111–117 loop; however, we do not observe complete opening of the C-terminus lid. In fact, the overall structure of the protein remains relatively similar to the bound crystal structure throughout. We do observe fluctuations in the 111–117 loop and the C-terminus that are important for product release.

Kinetic rate constants from simulations should be taken with a degree of skepticism as these are prone to large uncertainties. Small errors in the force fields or water model can lead to large errors in kinetic predictions from iMetaD simulations. Even with a deposition stride of 100 ps, metadynamics simulations can lead to errors in rate estimates if the bias is not along an optimal CV. For the CVs used here, the hyperdynamics time-rescaling approach gave a poor fit to a Poisson process with a *p*-value much less than 0.05. To alleviate this problem, we have used the recent Kramers’ time-dependent rate theory to fit numerical cumulative distribution times from the bias simulations. This has been shown to give a better rate estimate for the case of less-optimal CVs. Using this approach, we obtain rate estimates that are within the same order of magnitude as experiment. Transient kinetic analysis^8^ gives a rate for product release for 5-FU of 31 s^–1^, which is in surprisingly close agreement to our best fit value of 40 s^–1^. More informatively, the relative rate of product release for the uracil is predicted to be ∼10 times faster than for the 5-FU drug. It is interesting to compare this with the ratio of *k*_cat_ from experiments. The *k*_cat_ for cytosine to uracil is ∼5 times larger than *k*_cat_ for conversion of 5-FC to 5-FU,^8^ which compares well with our simulation, assuming that product release is the rate-limiting step.

In summary, this metadynamics study of the product release of 5-FU from the yCD enzyme could lead to future enzyme design that improves the prodrug/yCD catalytic efficiency by enhancing product release. For example, mutations could target the aromatic residues Phe114 and Trp152, residues in the 133–150 α-helix, or residues in the 111–117 loop, which are involved in the unbinding paths observed in our simulations. Our work also demonstrates how volume-based metadynamics and infrequent metadynamics can be used to study the thermodynamics and kinetics of drug release from a prodrug/enzyme system to provide a detailed picture of the drug release mechanism at the atomic scale.

## Data Availability

Input files to run volume-based MetaD and iMetaD simulations for both yCD/5-FU and yCD/uracil have been deposited to the public repository of the PLUMED consortium, PLUMED-NEST (plumID:24.008).

## Supporting Information Available

Depiction of the volumed-based CVs, the definition of the contact map CV used to construct the path CV in contact map space, the time-series trajectory of repeated 500 ns trajectories, the convergence of the calculated free energy difference in ligand unbinding, and the fit to the ECDF time from time-rescaled trajectories using the hyperdynamics approach (PDF)
