# Supplementary material for: Exploring Product Release from Yeast Cytosine Deaminase with Metadynamics
Source: J Phys Chem B. 2024 Mar 22;128(13):3102–12. doi: 10.1021/acs.jpcb.3c07972 (PMC11000218; doi:10.1021/acs.jpcb.3c07972)
Supplement: Supplementary file 1 — jp3c07972_si_001.pdf [file jp3c07972_si_001.pdf]

Supporting Information: Exploring Product Release from Yeast Cytosine Deaminase with Metadynamics

Kayla A. Croney and James McCarty*

Department of Chemistry, Western Washington University, Bellingham, WA, 98225, USA

E-mail: mccartj8@wwu.edu

This Supporting Information contains a depiction of the volumed-based CVs (Figure S1), the definition of the contact map CV used to construct the path CV in contact map space (Table S1), the time-series trajectory of repeated 500 ns trajectories (Figure S2), the convergence of the calculated free energy difference in ligand unbinding (Figure S3), and the fit to the empirical cumulative distribution time from time-rescaled trajectories using the hyperdynamics approach (Figure S4)

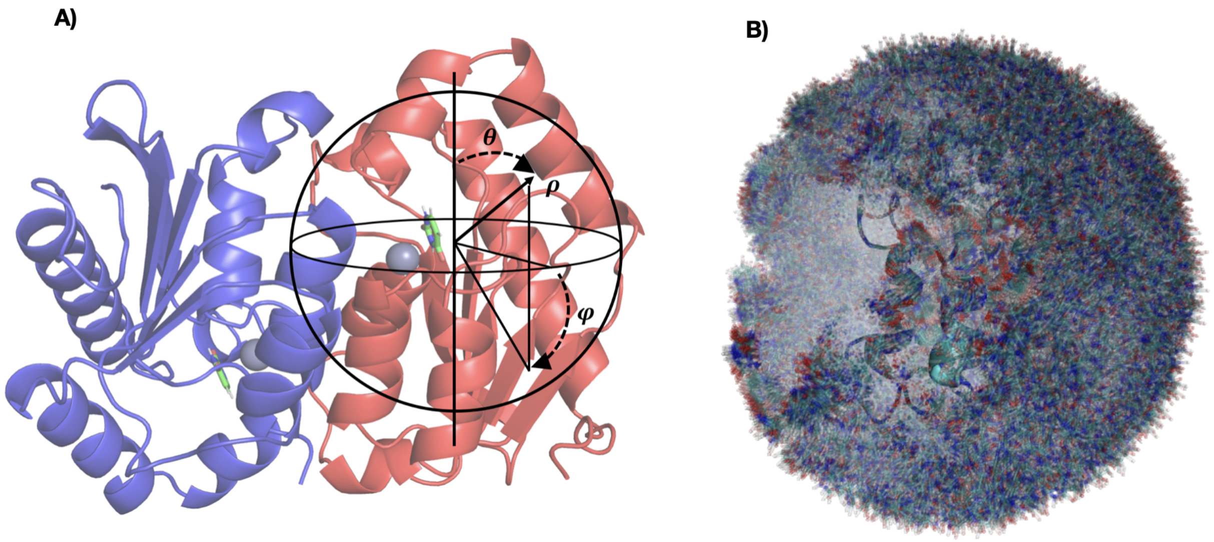

Figure S1: A) Schematic representation of the CVs used in a volumed-based metadynamics simulation. The spherical coordinates are ρ, θ, and ϕ, that define the position and orientation of the ligand relative to the binding site. B) An overlay of ligand configurations sampled during the 500 ns metadynamics simulation, showing complete sampling within the sphere defined by the restraining potential at 2.8 nm. The excluded volume region within the sphere is due to chain B of the homodimer not shown in the figure.

The contact map used to construct the path CV is defined as a vector with k-components being the contacts between atom pairs, calculated using a rational switching function of the form:

$$CM^{(k)} = \frac{1 - \left(\frac{r_{ij}}{r_0}\right)^n}{1 - \left(\frac{r_{ij}}{r_0}\right)^m} \tag{S1}$$

where r_{ij} is the distance between atoms i and j.

Table S1: List of atoms and parameters used in the contact map path CV.

Contact Number	Atom i	Atom j	r_0 [Å]	n	m
1	Ligand F (5-FU) or H (Uracil)	His62 C_β	12	6	12
2	Ligand F (5-FU) or H (Uracil)	Glu64 C_α	12	6	12
3	Ligand F (5-FU) or H (Uracil)	Phe114 C_β	12	6	12
4	Ligand F (5-FU) or H (Uracil)	Trp152 C_β	12	6	12
5	Ligand F (5-FU) or H (Uracil)	Zn^{2+}	12	6	12

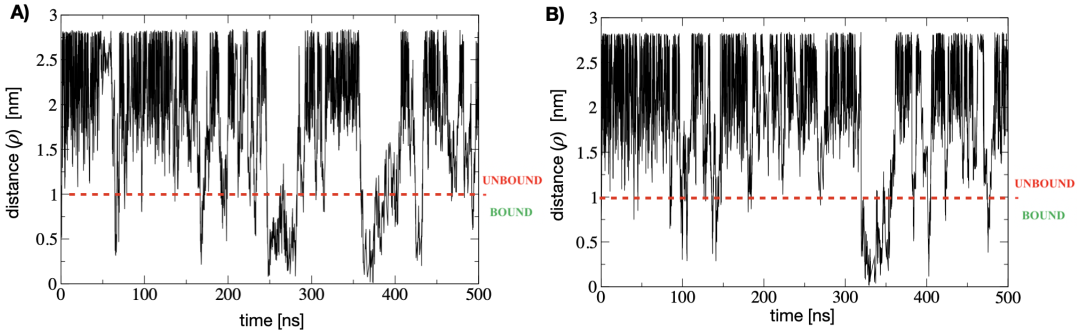

Figure S2: Time series of the distance between centers of mass of (A) 5-FU and (B) uracil from additional independent 500 ns metadynamics trajectories.

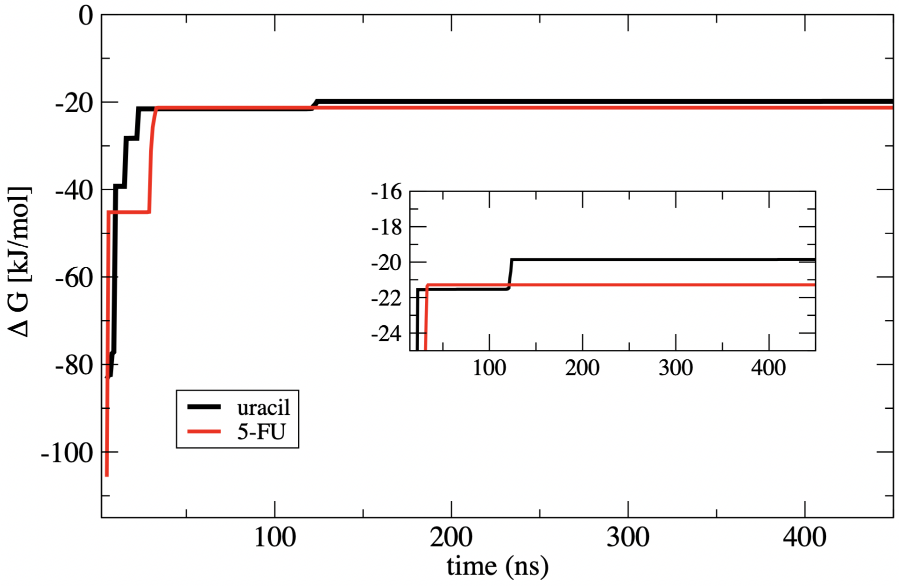

Figure S3: Time evolution of the value of ΔG_{MetaD} calculated from the reweighted free energy surface. The inset shows the final part of the convergence, showing that the estimate of ΔG has reached a stationary level.

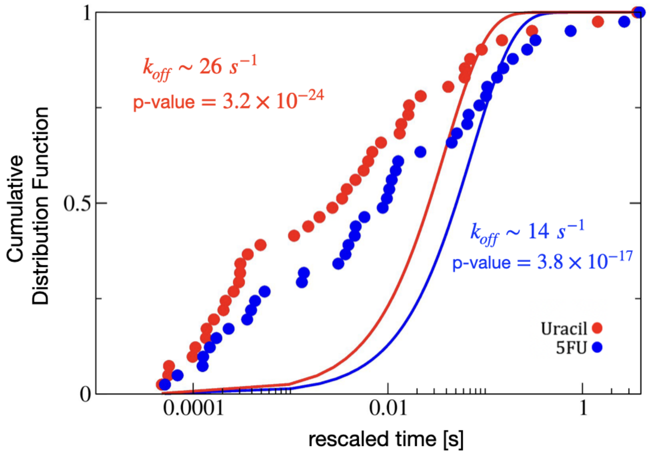

Figure S4: CDF of the iMetaD rescaled times using the hyperdynamics approach. Attempted fits are shown as solid lines. For these cases the KS- test fails as indicated by the p-value much less than 0.05.
